# Supplementary material for: Spatiotemporal structure of intracranial electric fields induced by transcranial electric stimulation in humans and nonhuman primates
Source: Sci Rep. 2016 Aug 18;6:31236. doi: 10.1038/srep31236 (PMC4989141; doi:10.1038/srep31236)
Supplement: Supplementary Information [file srep31236-s1.pdf]

Spatiotemporal structure of intracranial electric fields induced by transcranial electric stimulation in human and nonhuman primates – Supplementary Material

Alexander Opitz[1,2], Arnaud Falchier[1], Chao-Gan Yan[1,3], Erin Yeagle[4], Gary Linn[1,5], Pierre Megevand[4], Axel Thielscher [6,7,8], Michael P. Milham[1,2], Ashesh Mehta[4], Charles Schroeder[1,9]

Supplementary Material

EEG system and stimulator output testing

To rule out alternative explanations for the observed frequency response (see Supplementary Fig.1 and related description), we examined the frequency response of both the TES stimulator as well as the EEG system. We tested the EEG system by coupling a known oscillatory signal (using varying frequencies between 1Hz – 150 Hz) generated with a function generator between one recording channel and the reference channel. We found a small decrease in the recorded magnitudes (up to 10%) with increasing frequency for a constant amplitude input signal (S.1B). Similarly, we measured the output signal of the TES device measuring the voltage drop-off over a 5 kOhm impedance. We found only a slight decrease, maximal 3%, for the highest frequencies (S.1A). For both the stimulator output and EEG system, we fitted a 2nd degree polynomic curve for the frequency – magnitude response. A correction curve (S.1C) was obtained by multiplying both individual curves. The inverse of the correction curve was multiplied by the recorded voltages to correct for the dampening induced by the recording system for all measurements in the monkeys.

Saline Measurement

To test the recording setup and examine whether the frequency response observed in brain persists in a simple well-defined medium, we conducted one measurement in a saline solution using the same frequencies as for the monkey measurements. We recorded from 22 electrode contacts (two linear arrays) with 5mm spacing fixed inside the solution. Mean magnitudes showed a largely flat frequency response with a small decrease (5%) for high frequencies (S. 3A). Phase shifts were found to be around 2-4 degrees (S. 3B).

Effect of stimulation electrode type and recording order

In one session, we tested the effect of the type of stimulation electrode and the effect of the order of frequency recordings on the TES potentials. Recordings were performed as described in the methods section but in the first session, we used 8cm^2 sponge electrodes with Saline solution instead of gel electrodes. Right afterwards, we changed the stimulation electrodes to gel electrodes and repeated the recording session. In a final run, we repeated the same recording with a reversed order of frequencies using again gel electrodes. In general, we found only small differences between the different stimulation electrodes. Sponge electrodes showed a slightly steeper frequency drop-off (S. 4A) and slightly larger phase shifts than gel electrodes (S. 4B). The order of measurements of the randomized frequencies did not affect results. However, the first two measurement points after changing from Sponge to Gel electrodes resulted in enhanced magnitudes likely resulting from quickly changing electrode impedance, until electrode impedance stabilized.

Effect of reference electrode position

In one session, we tested the influence of the position of the reference electrode. For that we recorded potentials from 5 Hz tACS using the same stimulation electrode positions as during the main experiments and a stimulation intensity of 500 muA. We varied the position of the reference electrode from near the right ear (A), vertex (B), to the left temple (C) and recorded 30s of data for each reference electrode. The position of the reference electrode has a marked influence on the amplitude and shape of the recorded potentials (S. 5A-C, left panel) with phase reversals occurring for references A and C and some clipping for one channel for reference B. Nevertheless, the relative spatial distribution of potentials is identical irrespective of reference position (S. 5A-C, right panel). Similarly, the reference position also has a marked influence on the magnitude of the recorded potentials at various channels (S. 6A) as well as the mean magnitude over all channels (S. 6B). These results are not surprising as the reference electrode also picks up the stimulation current and will thus exhibit, depending on position, a smaller or larger potential value. This will affect recorded potentials as the difference between the potential at the electrode contact and the reference signal. An electric potential is only defined up to a constant value, thus its absolute value is not physically meaningful. However, differences between potentials (the electric field) are physically meaningful. Indeed computing difference signals between all electrode contacts leads to identical magnitudes, irrespective of reference electrode position, both for individual contact differences (S. 7A) as well as their mean values (S. 7B). Computing a frequency-magnitude relation for the difference signals from the measurement data used in the main analysis leads to a very similar relationship, a small decrease of magnitude with increasing frequency for both monkeys (S.8 A+B). Small differences in the magnitudes between repeated measures induced by baseline shifts or changes in electrode impedances might even be slightly reduced by using difference signals. For the analysis of phase relations, we note that a phase relationship of π (180 degree) still represents an in-phase signal. Thus, for the phase analysis we computed phase differences modulo π and not 2π as is standard. Altogether the exact position of the reference does not seem to significantly affect results and could be thus chosen based on experimental considerations. From a practical perspective the choice of reference electrode position should optimize (1) capturing the recorded signal within the dynamic range of the recording system, (2) accessibility on the head and (3) distance from possible noise sources.

Raw data

Exemplary raw data for Patient 1 is shown in Supplementary Figure 9 (1Hz stimulation). Raw data for Patient 2 is displayed in Supplementary Figure 10 (1Hz stimulation). High frequency potentials (100 Hz) for Monkey 1 are shown in Supplementary Figure 11.

Intensity sweep

While the primary focus of the present work is on phase effects, we did carry out an intensity sweep between 50 µA and 500 µA at the beginning of one of the recording sessions to test the linearity between stimulation currents and recorded voltages included. We found that the magnitude of recorded voltages increased linearly with current strength (S.12 A) as expected by Ohm's law. Phase differences were found to be small irrespective of current strength (S. 12 B).

This highlights the ability of our measurement setup to accurately capture electric field properties irrespective of the chosen current strength.

Spatial Distribution of tACS phases

The phase of tACS-induced potentials across contacts is displayed in Supplementary Figures 13 and 14 for both monkeys and patients. A small change of phase was found with increasing distance from the stimulation electrodes for both monkeys. The findings in patients were generally similar to those in monkeys, though some electrodes exhibited incidentally higher phase differences (S. 14). These were only present at electrodes with very low signal amplitude, which does not allow accurate phase estimation. At contacts with higher signal amplitudes, very small phase differences were found as in the monkey recordings.

Anatomical locations of recording electrodes

The locations of recording electrodes in their anatomical MR images for Monkeys 1 + 2 are shown in Supplementary Figure 15. The CT images of patients 1 + 2 with the implanted electrodes are shown in Supplementary Figure 16.

Supplementary Figure 1: Calibration measurements of the frequency dependency of the electrical stimulation device and EEG system. **A)** Normalized magnitude of the output of the stimulation device. A minimal decrease in stimulation intensity was found with increasing stimulation frequency (3% decrease for 150 Hz). **B)** Frequency response of the EEG system. A slight decrease in magnitude was found with increasing frequency with a 10% decrease at 150 Hz. **C)** Calibration curve to correct for frequency dependencies induced by the experimental equipment. Stimulator output and EEG response were treated as two independent multiplicative factors to generate a correction factor for the measurement results.

Supplementary Figure 2: Magnitude and phase analysis of tACS potentials. **A)** Example recording from one electrode (12 contacts) for a stimulation frequency of 10 Hz (left panel). Signals are strongly phase aligned to each other. A narrow power spectrum for the 10 Hz stimulation frequency without the presence of harmonics is visible for all channels (right panel). **B)** FFT magnitude at one selected channel with increased resolution again showing the narrow frequency peak (left panel). Histogram of phase differences (right panel) for the 10 Hz example shows the small differences between channels.

Supplementary Figure 3: Bode plot illustrating the frequency dependency of magnitude and phase differences of TES induced electric potentials measured in a saline solution. **A)** Normalized mean magnitude over all contacts in dependence of stimulation frequency (log units) from 1Hz – 150 Hz. A nearly flat frequency-magnitude response with a slight decrease (5%) at high frequencies is visible. **B)** Mean phase differences (degree) between all combinations of electrode contacts. Small phase shifts within a few degrees were present for all measured frequencies.

Supplementary Figure 4: Impact of order effects during the measurement and effect of stimulation electrodes. **A)** Normalized mean magnitude over all contacts in dependence of stimulation frequency (log units) from 1Hz – 150 Hz for three measurements using sponge

electrodes, gel electrodes and gel electrodes with a reversed order of measurement frequencies. Small differences were found between sponge and gel electrodes with sponge electrodes showing a slightly more steep decrease with higher frequencies. The order of recorded frequencies did not affect the measurement results. Note however, the two enhanced measurement results for the first gel measurement. These two points correspond to the first two measured frequencies after switching from sponges to gel electrodes with a quick change in the impedance of the stimulation electrodes until a stable impedance value was reached. Thus changes in electrode impedance can significantly affect stimulation strength and should be taken into account. **B)** Mean phase differences (degree) between all combinations of electrode contacts for the three measurements. Small effects of stimulation electrodes and recording order were found for the phase differences with slightly larger phase shifts for sponge electrodes.

Supplementary Figure 5: Effect of placement of the reference electrode on recorded potentials. Recorded potentials are shown for all electrode contacts for a few stimulation cycles (left panel) as well as their spatial distribution for the reference electrode placed near the right ear **A)**, near vertex **B)**, or left temple **C)**. The amplitude and absolute value of the recorded potentials markedly differ depending on the position for the reference electrode. Nevertheless, their relative spatial distribution is unaffected by the choice of reference electrode. Of note is the possibility of clipping artifacts (B) and possible phase reversals depending on reference electrode position.

Supplementary Figure 6: Effect of placement of the reference electrode on the recorded potentials. **A)** Magnitude of recorded potentials for the three different reference positions for all electrode contacts. A clear dependency on the position of the reference electrode is visible on potential magnitude over the different recording contacts. **B)** Mean normalized magnitude over all electrode contacts for the three reference positions for two measurements each. Depending on the position of the reference electrode, mean magnitude can differ significantly.

Supplementary Figure 7: Effect of placement of the reference electrode on the potential differences. **A)** Magnitude of difference signals between all possible contact combinations for the three different reference positions. Using difference signals, all measurements lead to nearly identical results irrespective of the position of the reference electrode. **B)** Mean normalized magnitude over all electrode contact differences for the three reference positions for two repeated measurements. Using difference signals, all three positions of the reference electrode lead to nearly identical results.

Supplementary Figure 8: Frequency-magnitude plots showing normalized mean difference magnitudes over all contact combinations for Monkey 1 **A)** and Monkey 2 **B)** A similar relationship as for potential magnitudes was observed (see Figs. 1+2) with a slight decrease in magnitude with increasing frequencies. Differences between measurements likely due to small changes in electrode impedance are slightly reduced using difference signals.

Supplementary Figure 9: Demonstration of baseline correction procedure. Measurement data from Patient 2 are shown. Onset of tACS takes place at around 7s. **A)** Recorded potentials for

three adjacent channels are shown before baseline correction. A DC offset is visible between channels. **B)** Potentials after BL correction removing DC shifts between channels.

Supplementary Figure 10: Example of raw data for Patient 1. Onset of tACS takes place at around 0s. **A)** Recorded potentials for three adjacent channels are shown. Potentials from different channels are closely phase aligned. **B)** Measurement data across all channels (Potential Amplitude color coded). Alignment of peak and troughs across different channels is visible.

Supplementary Figure 11: A). Exemplary raw data of tACS potentials recorded for high frequency (100Hz) stimulation (sampling rate 500 Hz).

Supplementary Figure 12: A) Intensity sweep demonstrating the linearity between stimulation currents and recorded potentials (Ohm's Law). Data recorded in Monkey 2. Shown is the magnitude of recorded potentials for different current intensities (50 µA to 500 µA) measured at 1Hz. **B)** Estimation of phase differences for different current intensities measured at 1Hz. Phase differences were found to be independent of recording intensity.

Supplementary Figure 13: Spatial variation of the phase (in degree) of recorded potentials during tACS (1Hz) in the monkeys. **A)** Monkey 1: A small systematic shift in phase is visible along the recording contacts. **B)** Monkey 2: Similar to Monkey 1 small phase changes are visible between recording contacts.

Supplementary Figure 14: Spatial variation of the phase (in degree) of recorded potentials during tACS (1Hz) in the patients. **A)** Patient 1: While a majority of recording contacts exhibit similar phase relationships some contacts have larger deviating phases. These deviations occur at contacts with small recorded potentials that do not allow for accurate phase estimation (see example in B). Smaller potentials are due to the proximity to the reference electrode. **B)** Patient 2: Phase distributions showing majority of contacts exhibit similar phase. Contacts with large deviating phase occur at locations with very low signal. Right: Example of recorded potentials of contact with low signal resulting in unreliable phase estimation (blue trace) in comparison to contact with good signal and reliable phase (green trace).

Supplementary Figure 15: Anatomical MR images of the monkeys with implanted electrodes for Monkey 1 **A)** and Monkey 2 **B)**. Displayed are sagittal MR cuts showing the susceptibility artifacts of the implanted electrodes on the anatomical image indicating the anatomical location.

Supplementary Figure 16: CT images of the patients with implanted stereo-EEG and grid electrodes. **A)** Two coronal MR cuts of Patient 1 with bilateral implanted stereo-EEG electrodes are shown. **B)** Patient 2 with left hemispheric grid electrodes and stereo-EEG electrodes shown in one coronal (left) and sagittal (right) cut.

Supplementary Figure 17: Frequency-magnitude plots showing normalized mean magnitudes over all contacts on a linear scale for Monkey 1 **A)** and Monkey 2 **B)** with a linear fit for the observed small decrease in magnitude with higher frequencies.

Supplementary: Calibration measurements

Supp Fig1

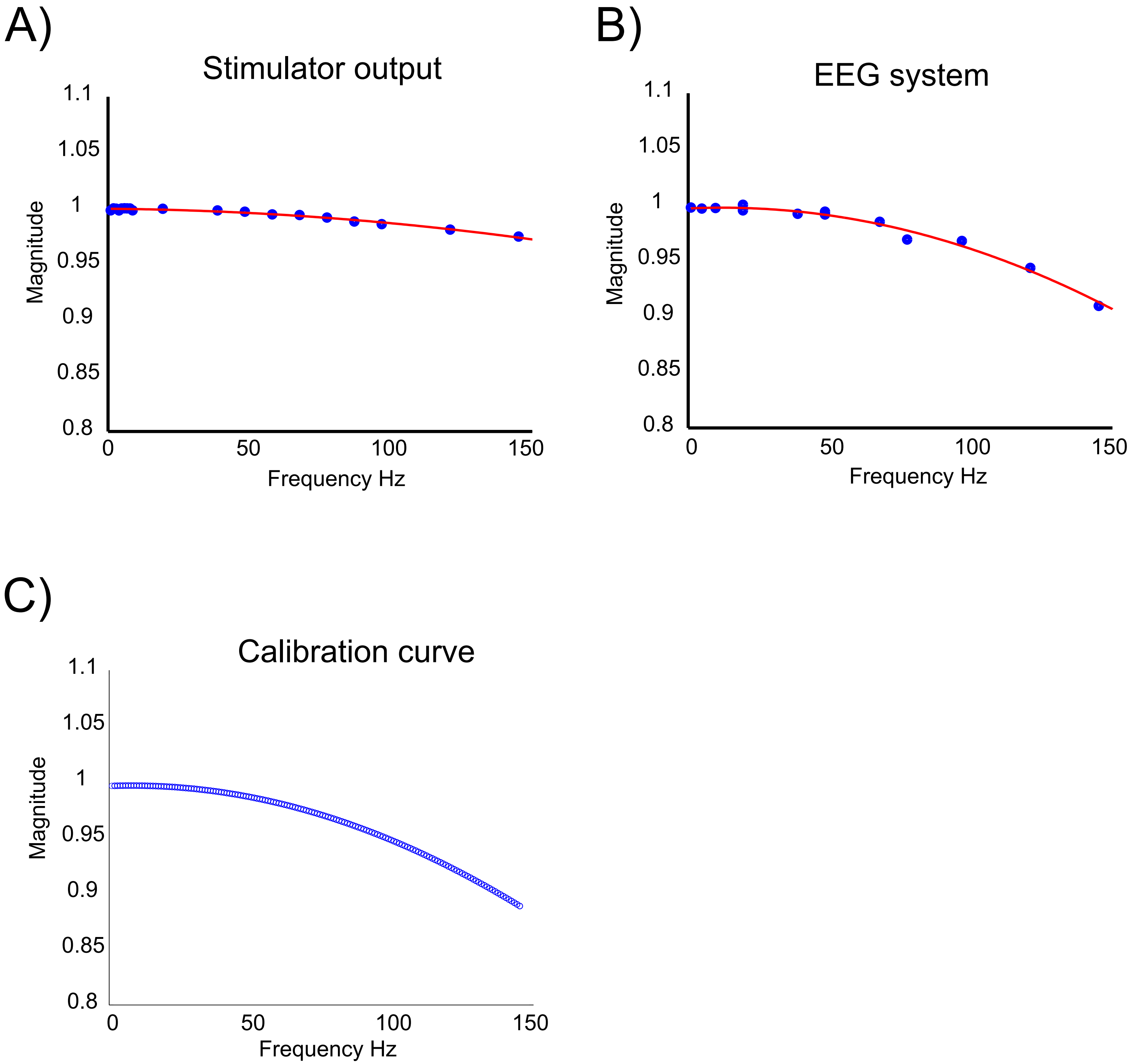

Supp Fig2

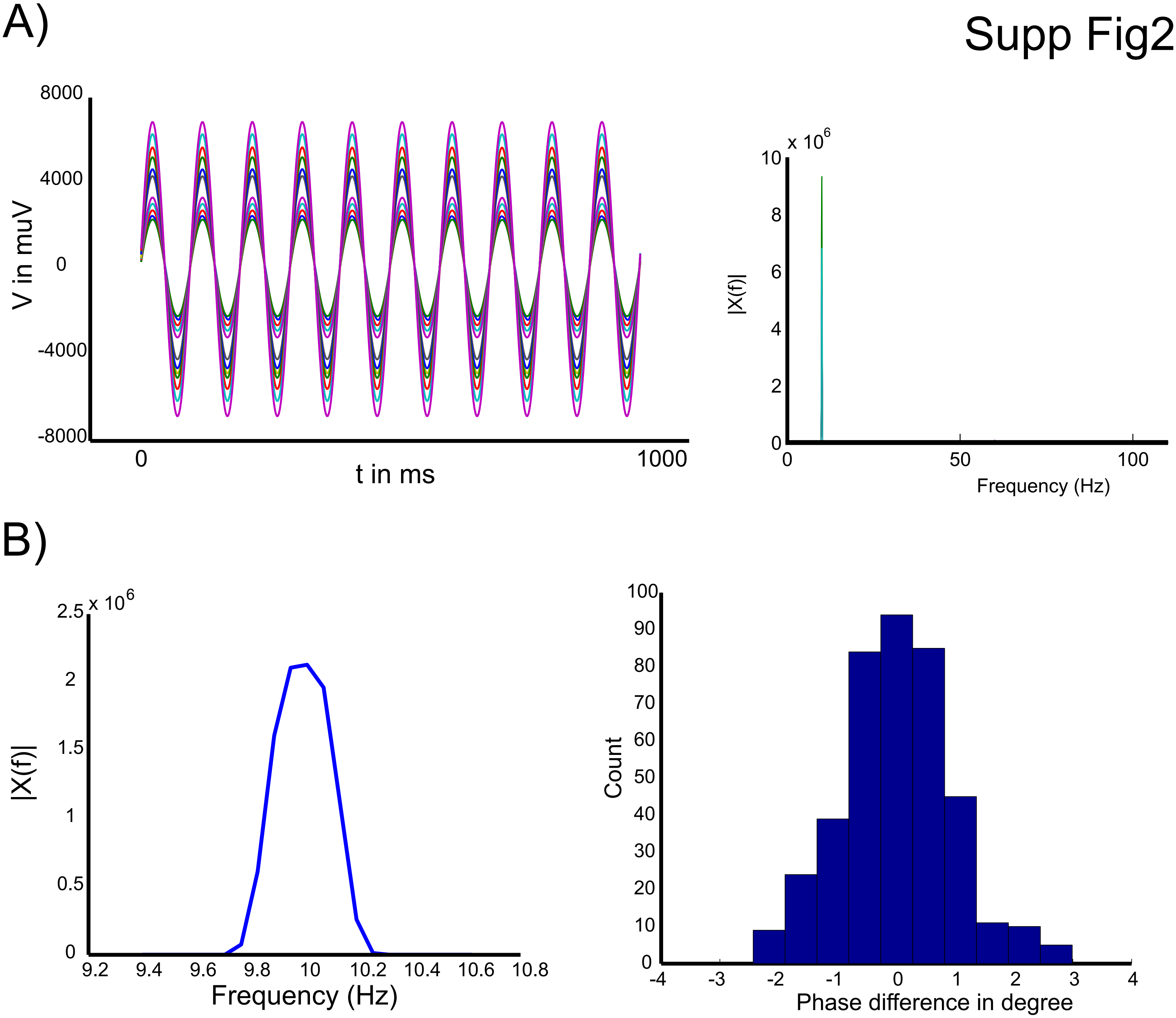

A)
8000
4000
0
-4000
-8000
V in muV
0
1000
t in ms
10
x 10^6
8
6
4
2
0
|X(f)|
0
50
100
Frequency (Hz)
B)
2.5
x 10^6
2
1.5
1
0.5
0
|X(f)|
9.2
9.4
9.6
9.8
10
10.2
10.4
10.6
10.8
Frequency (Hz)
100
90
80
70
60
50
40
30
20
10
0
Count
-4
-3
-2
-1
0
1
2
3
4
Phase difference in degree

Saline Measurement

Supp Fig3

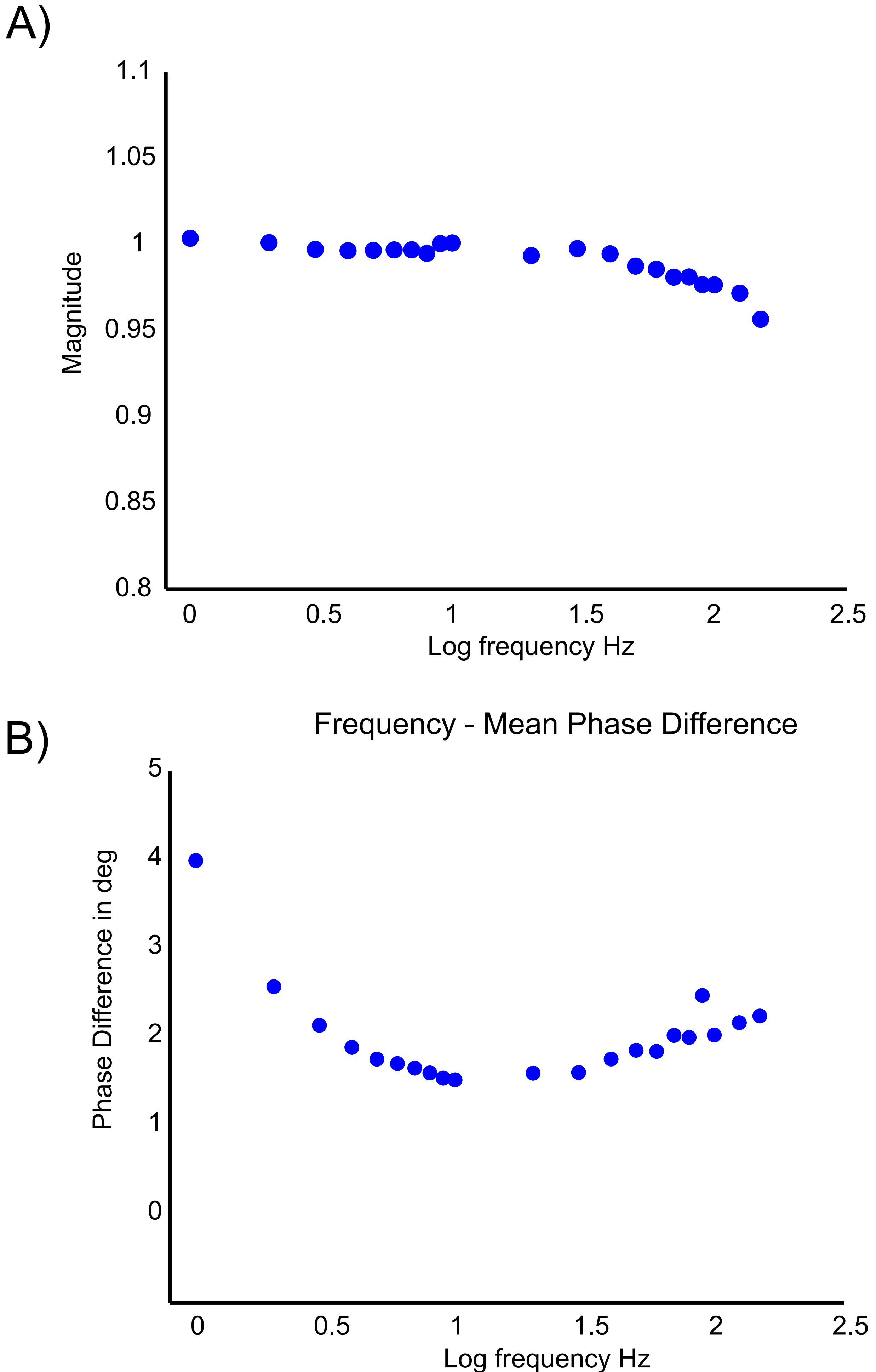

Order effects + Gel vs Sponge

Supp Fig4

A)

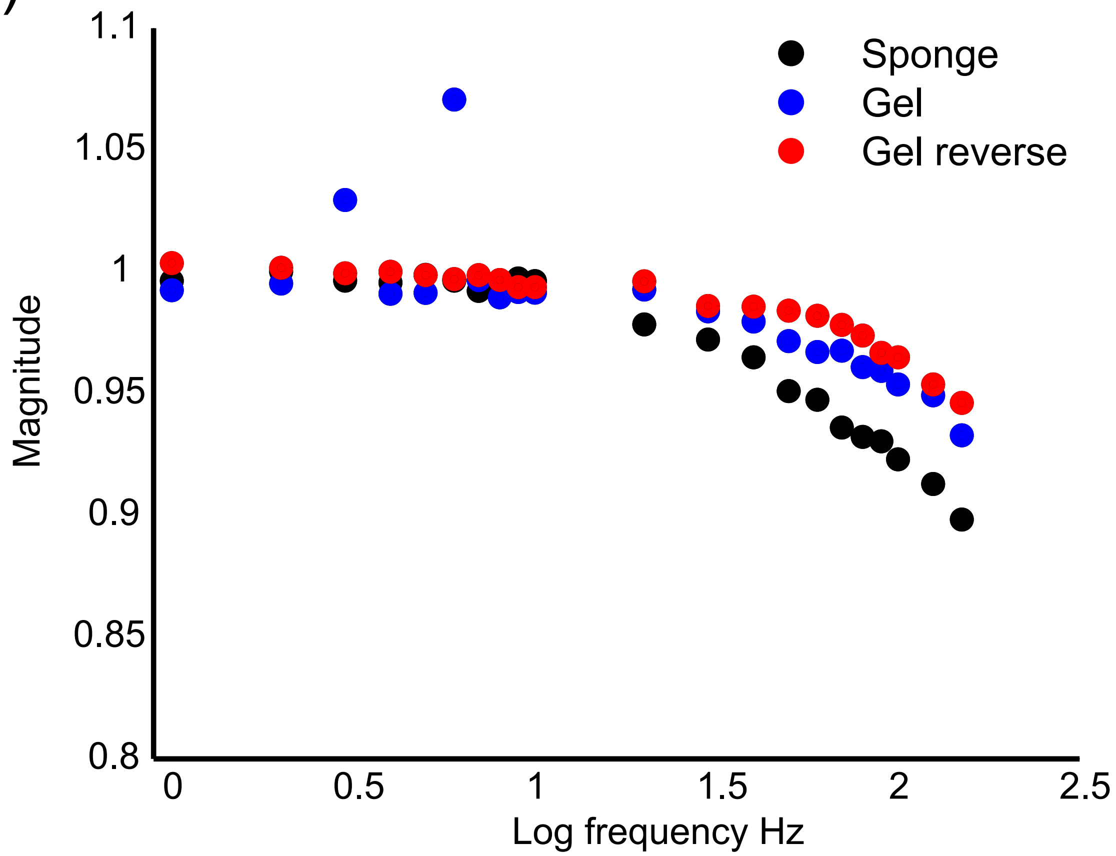

B)

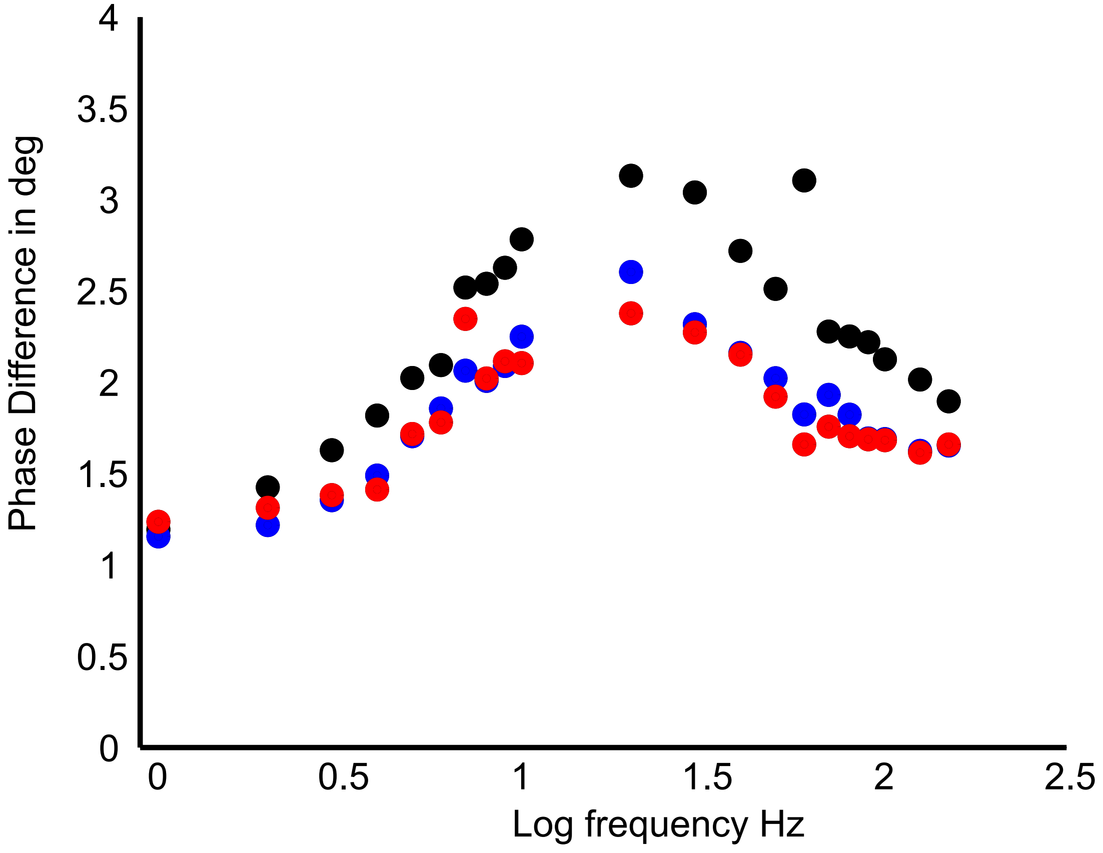

Supp Fig5

Reference electrode placement

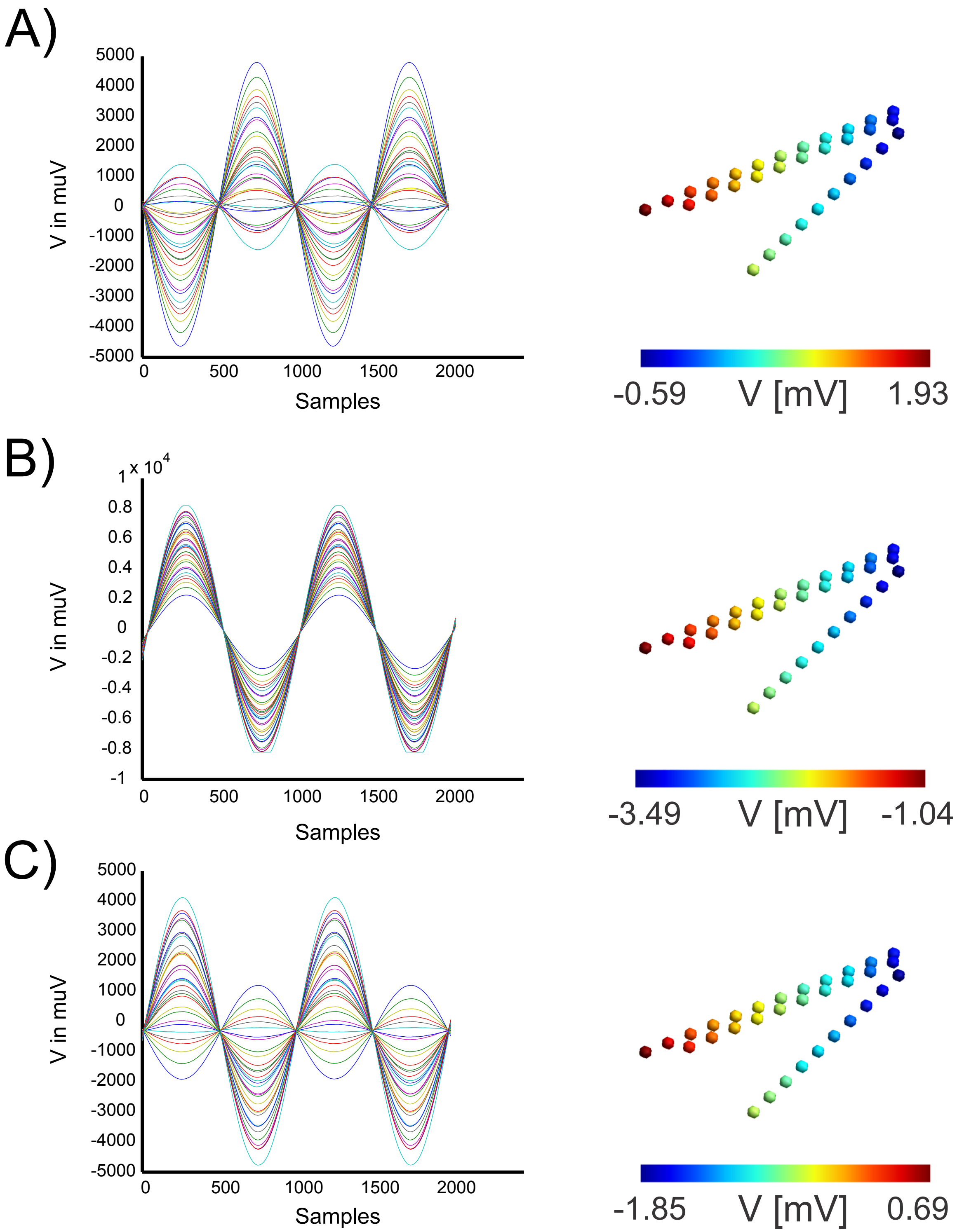

Reference electrode placement

Supp Fig6

A)

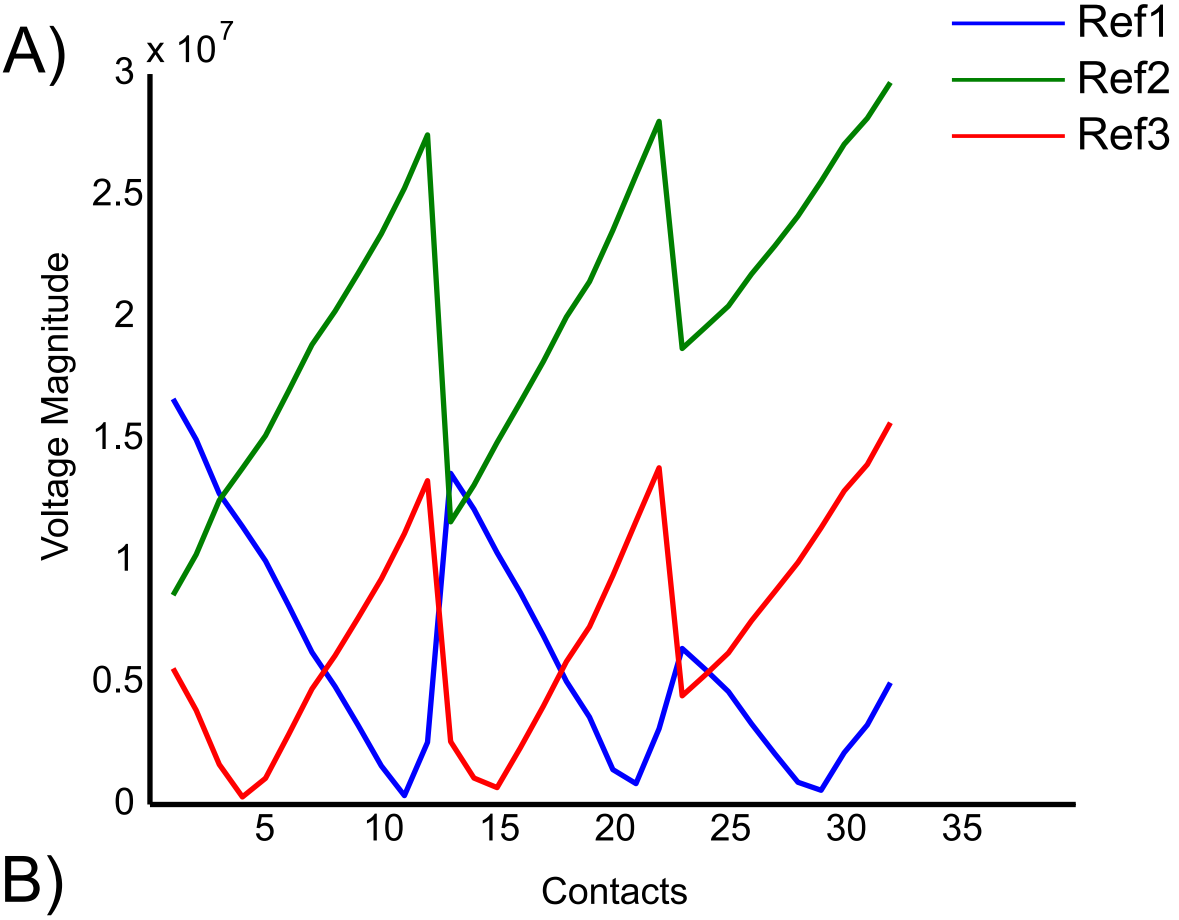

B)

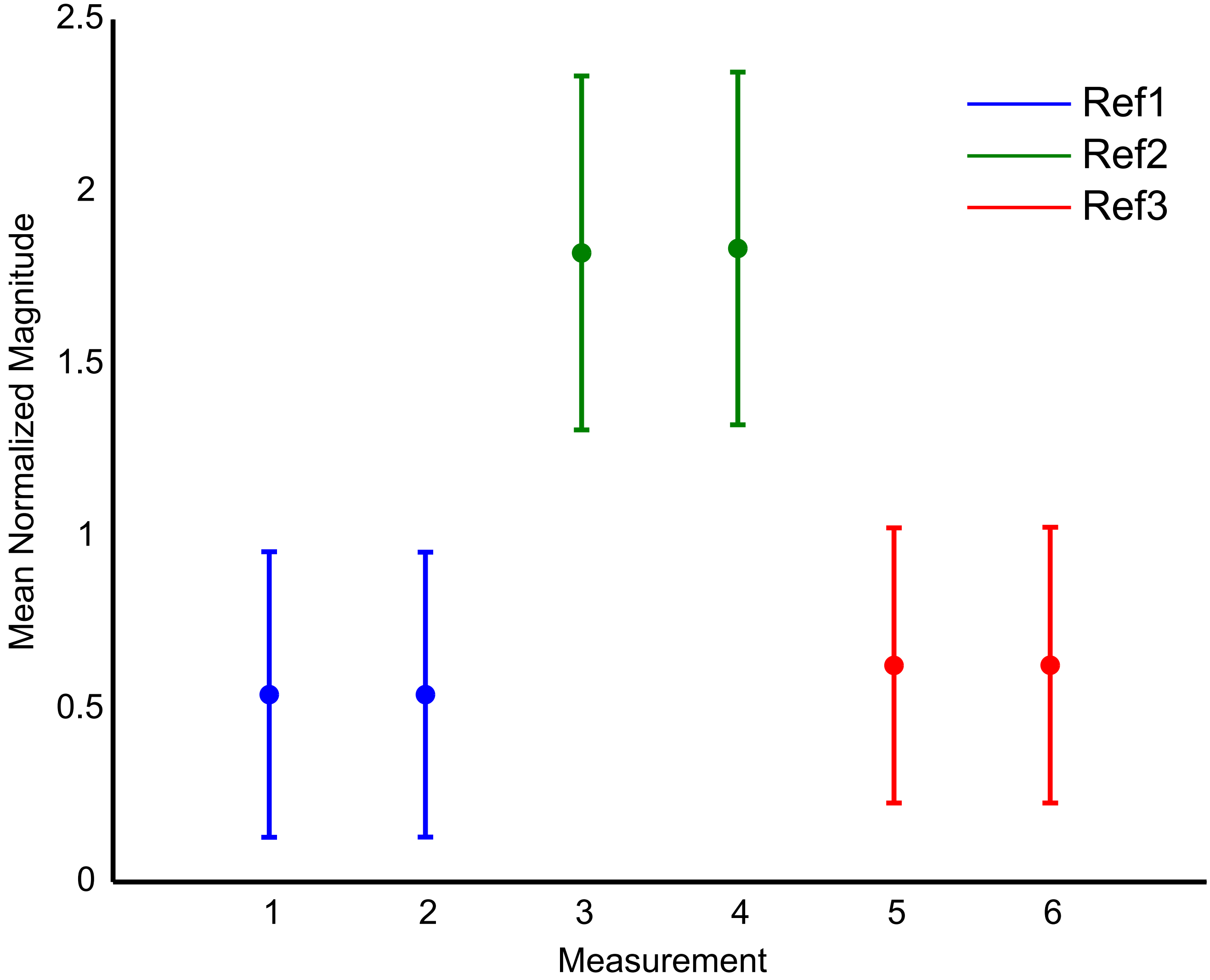

Reference electrode placement

Supp Fig7

A)

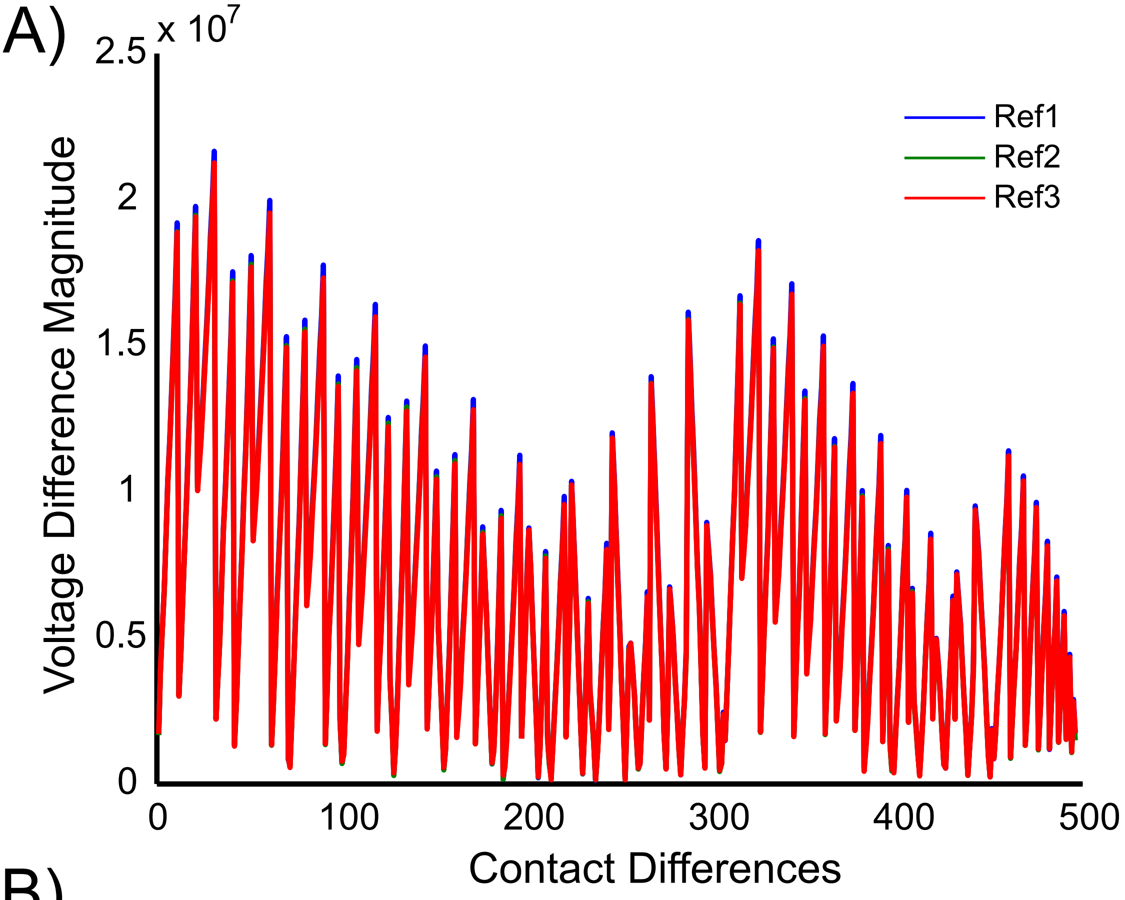

B)

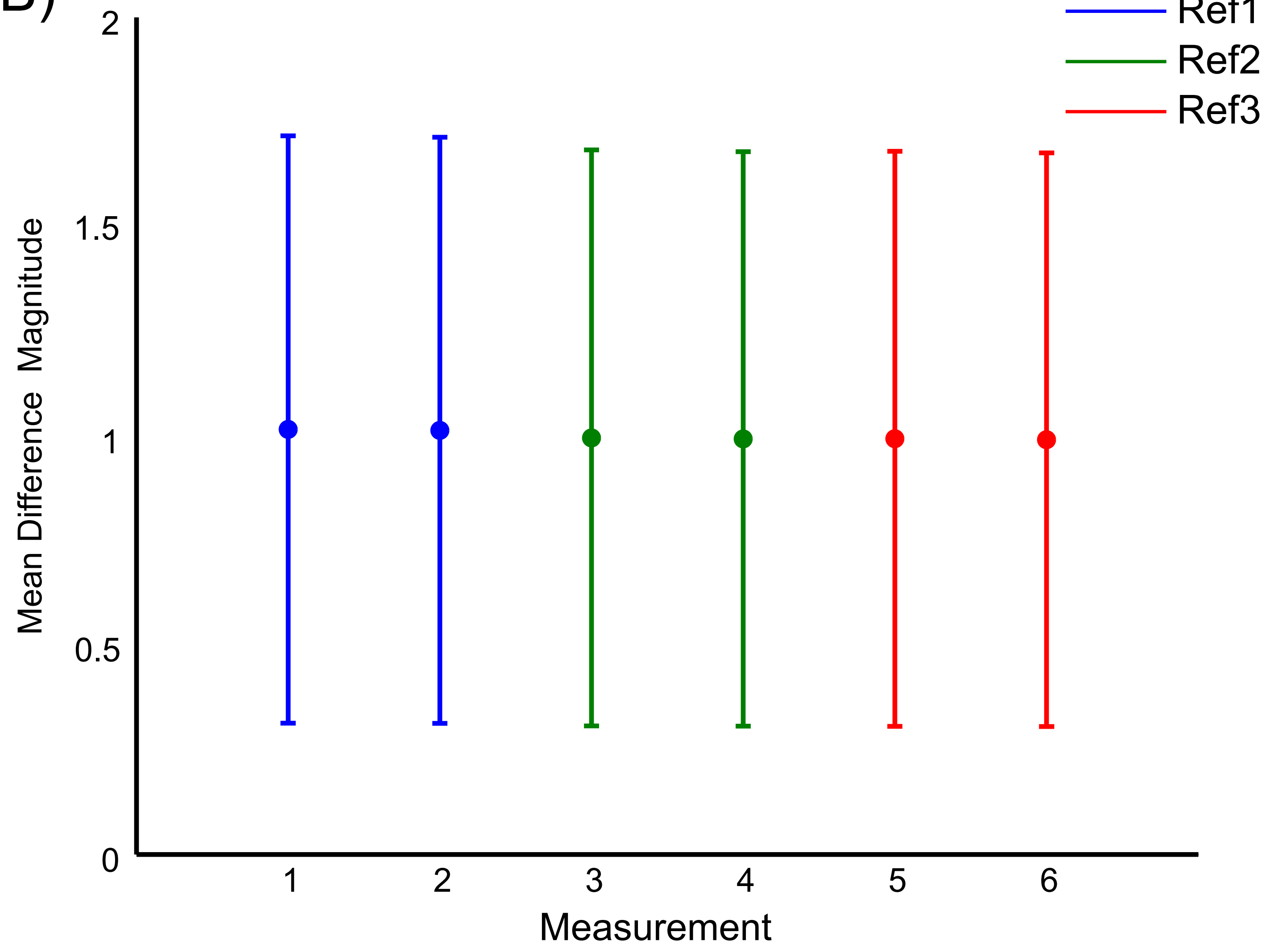

Supp Fig8

Difference signals

A)

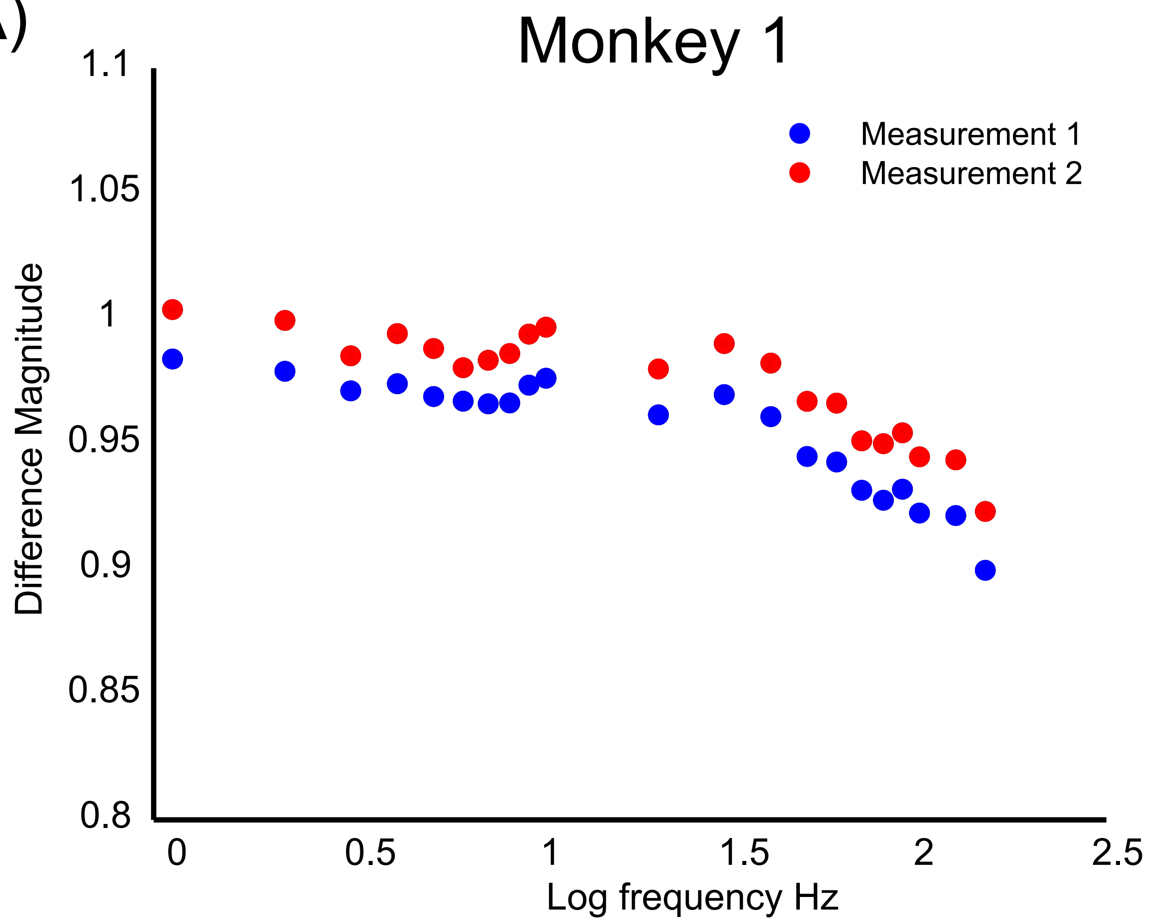

B)

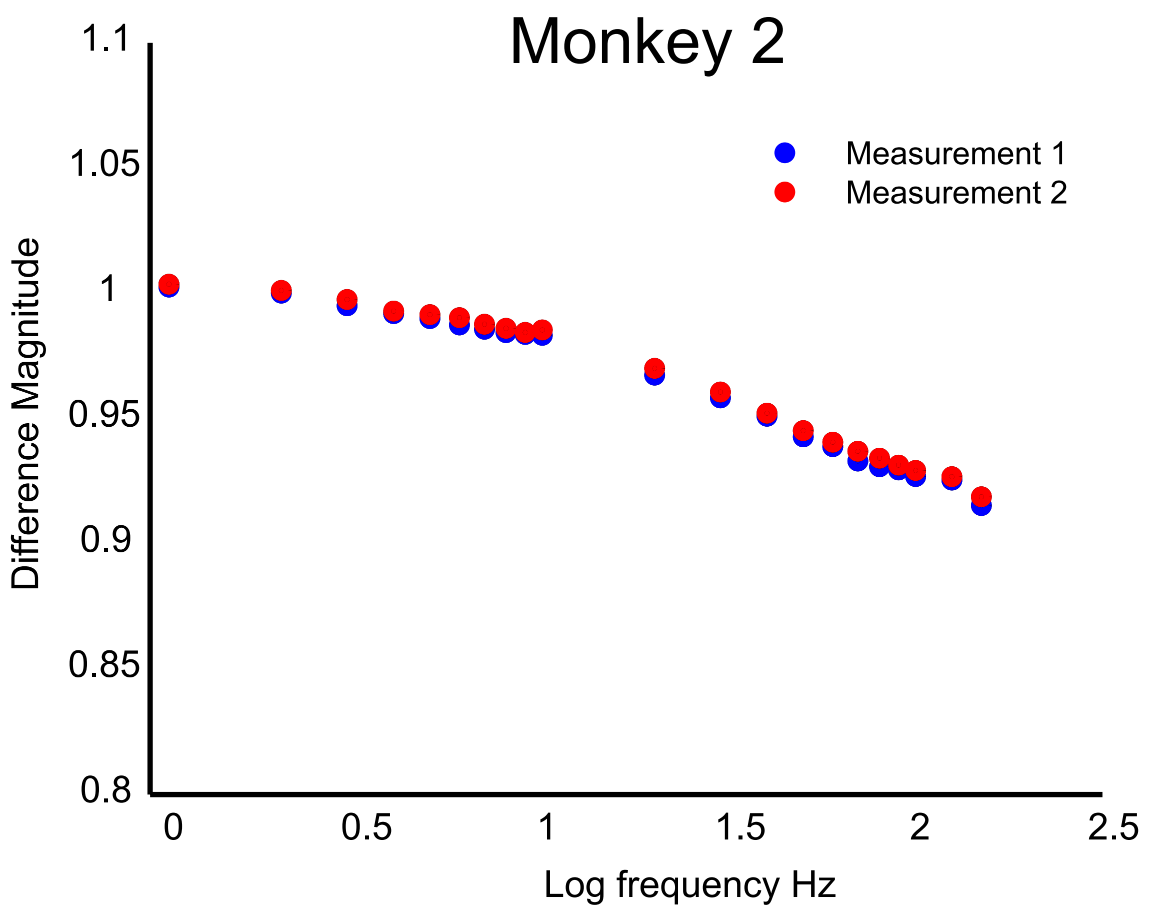

Supp Fig9

A)

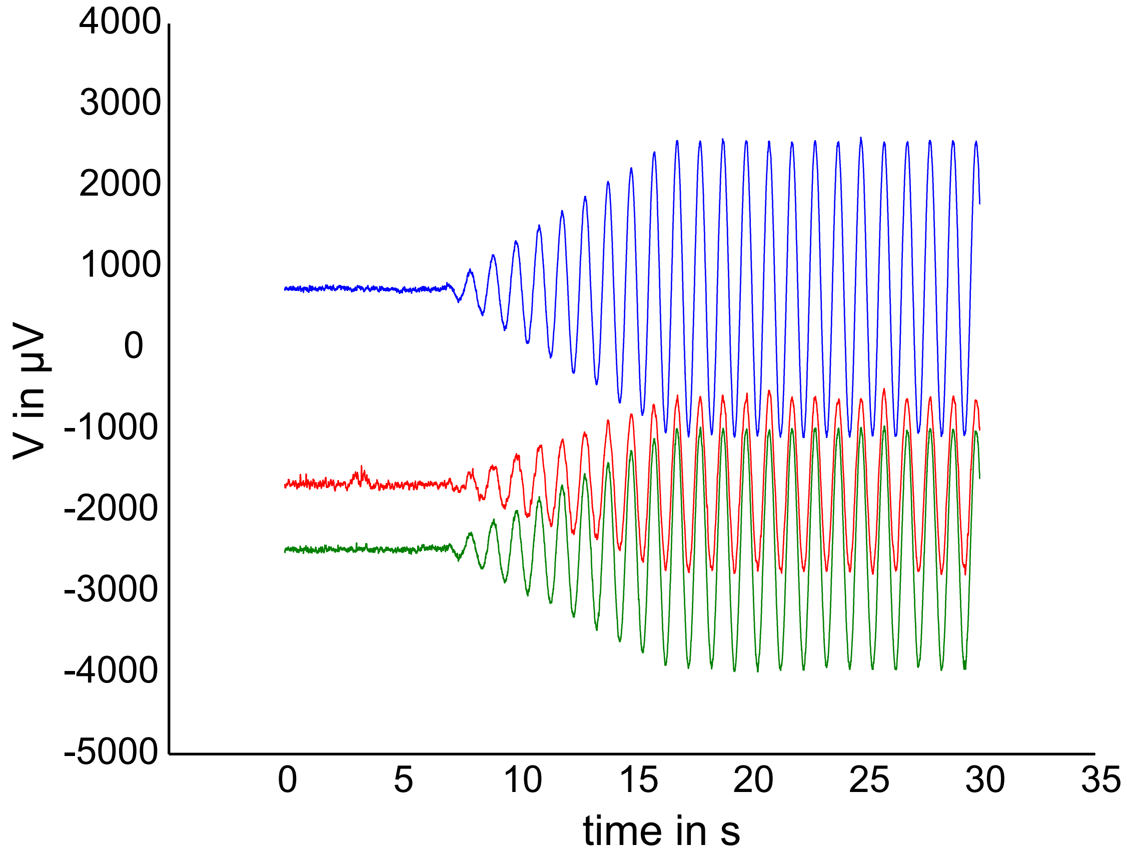
4000
3000
2000
1000
0
-1000
-2000
-3000
-4000
-5000
V in µV
0
5
10
15
20
25
30
35
time in s

B)

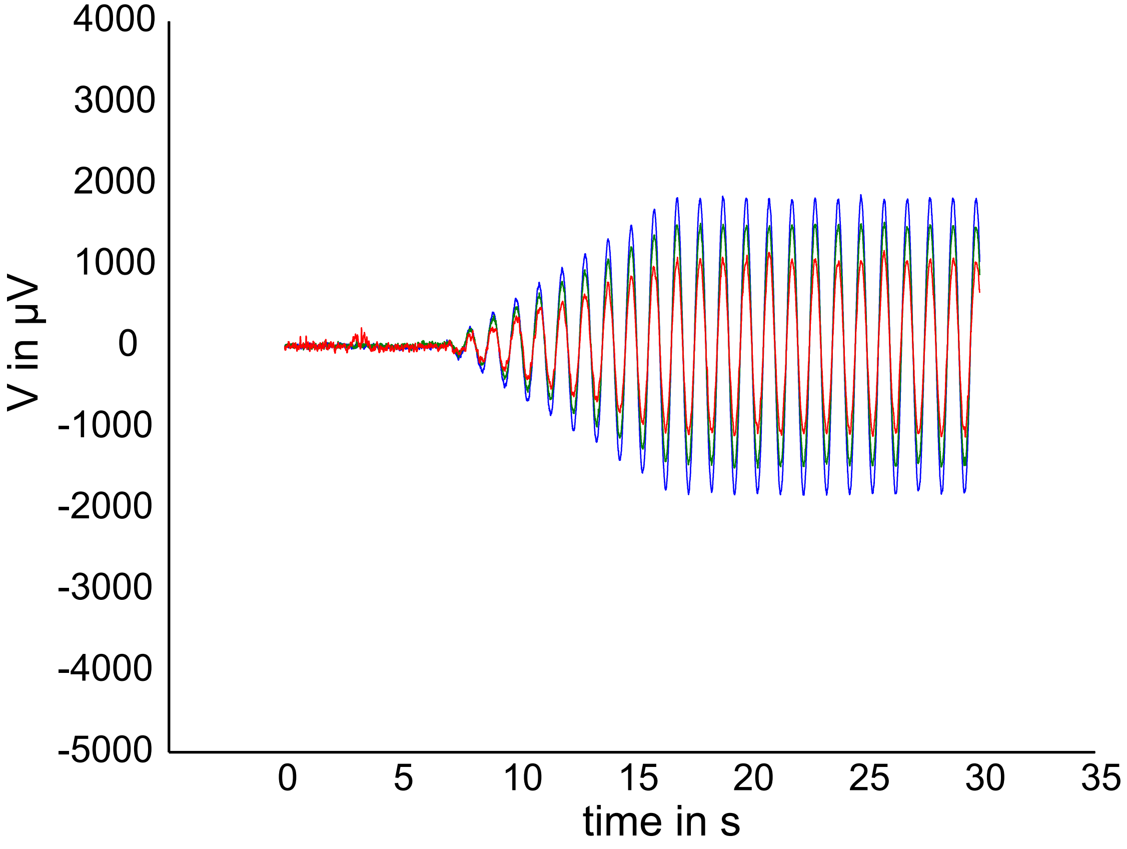
4000
3000
2000
1000
0
-1000
-2000
-3000
-4000
-5000
V in µV
0
5
10
15
20
25
30
35
time in s

Supp Fig10

A)

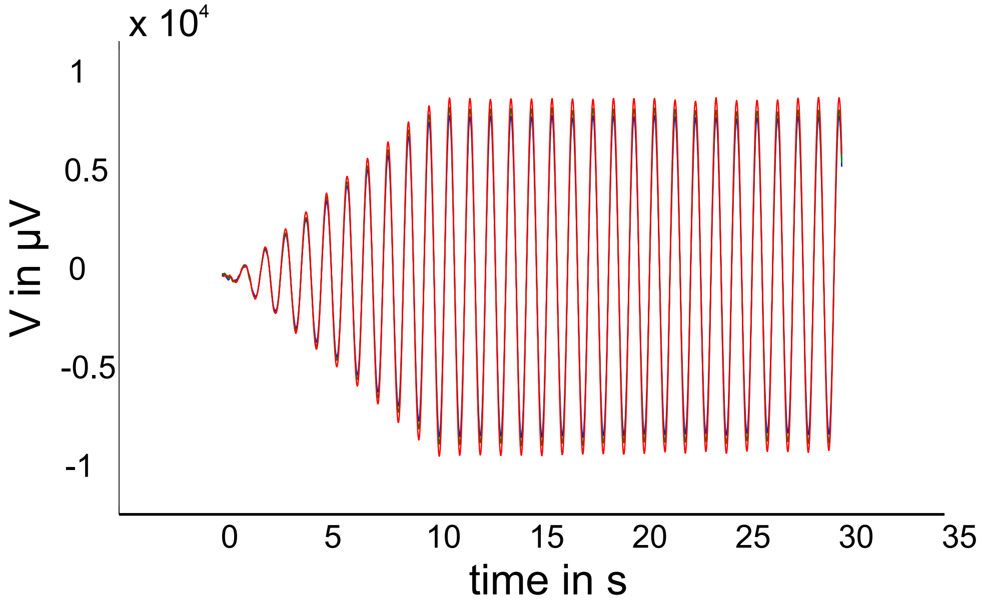

B)

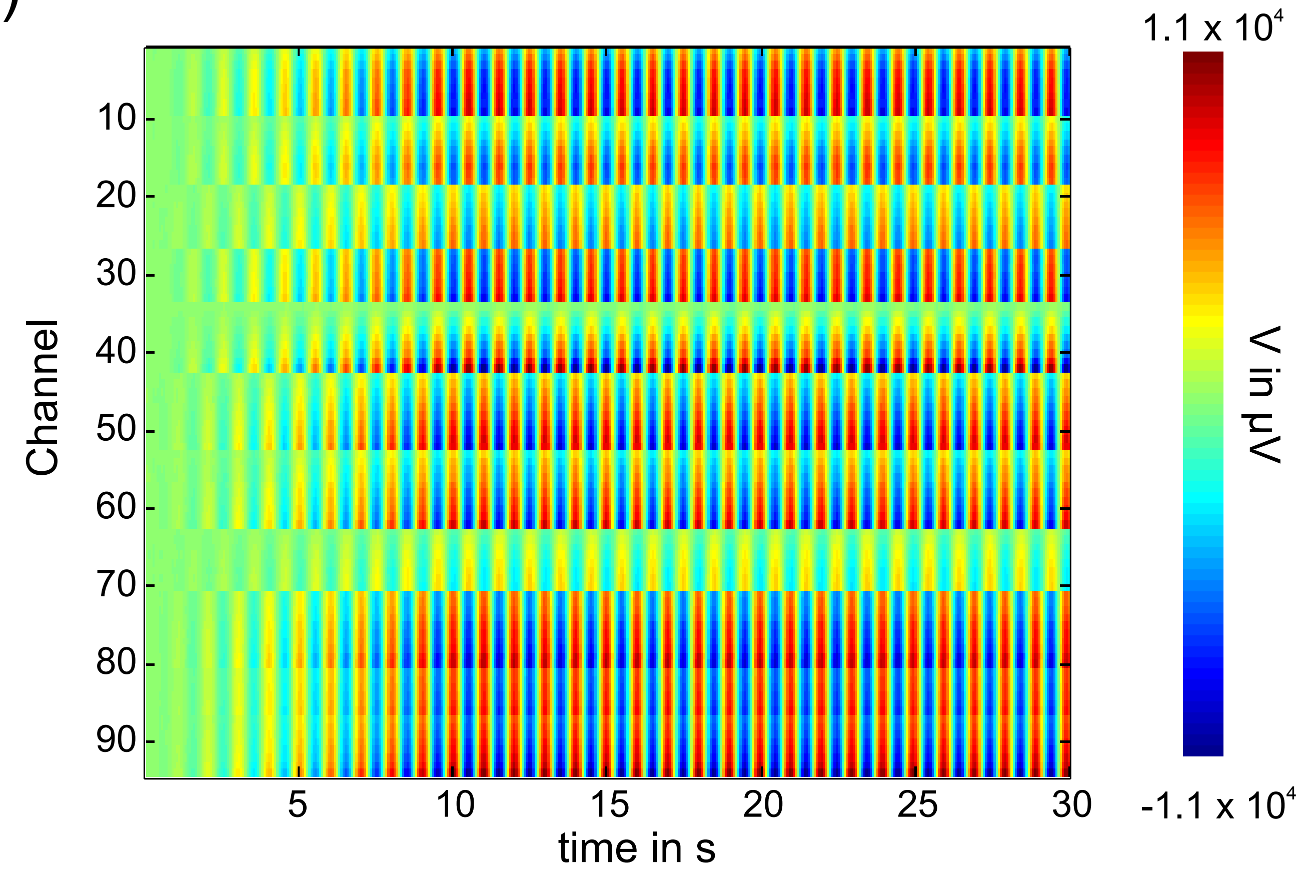

Supp Fig11

A)

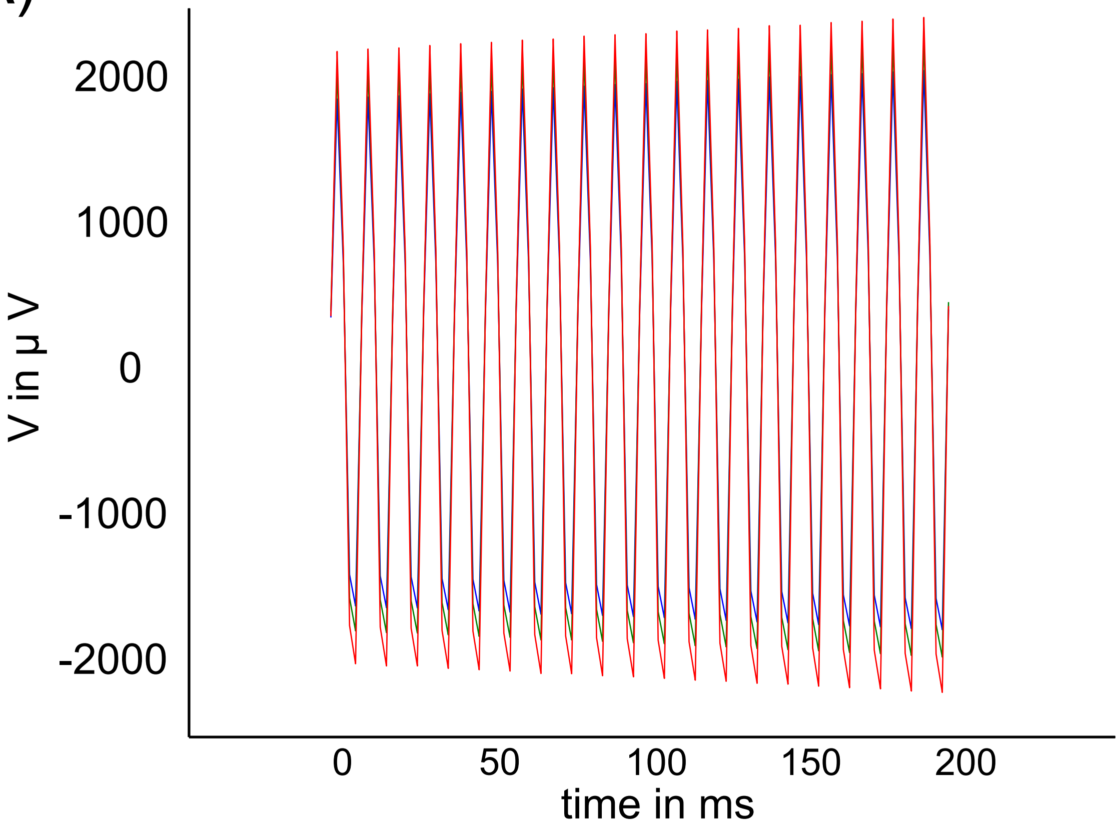

A)

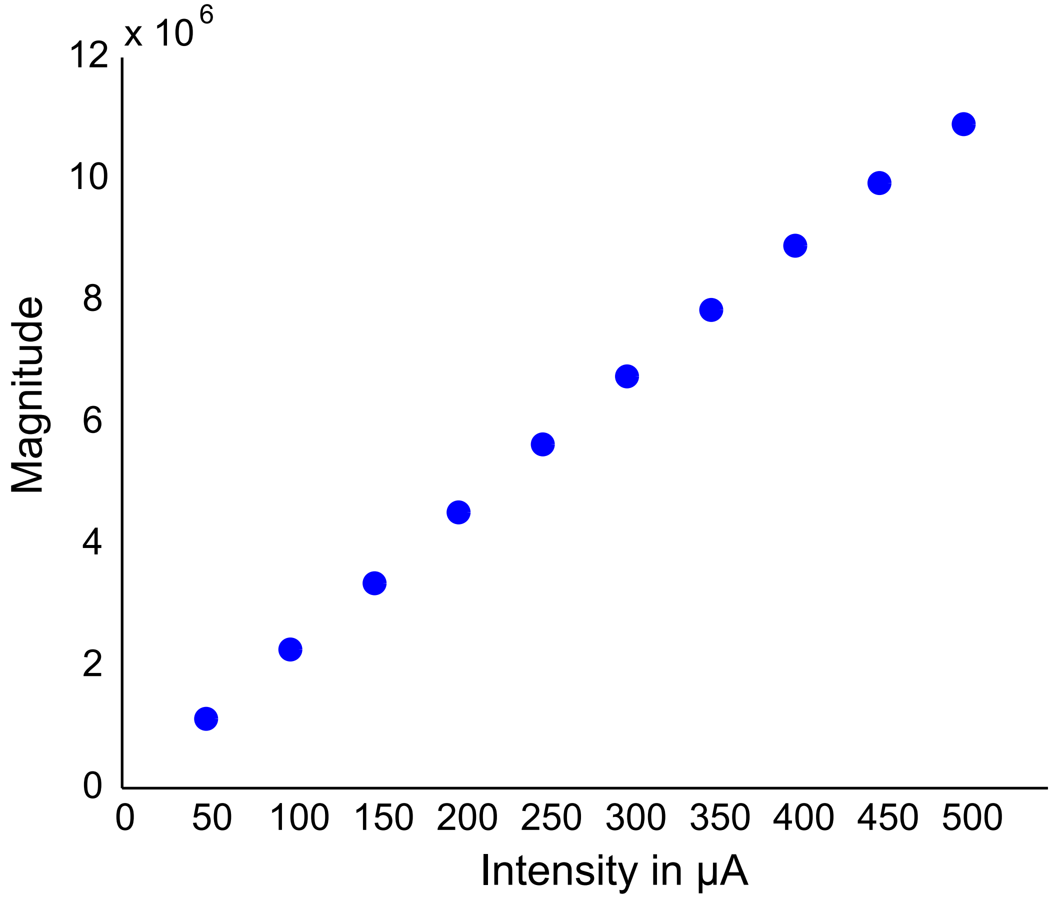
x 10^6
12
10
8
6
4
2
0
Magnitude
0
50
100
150
200
250
300
350
400
450
500
Intensity in µA

B)

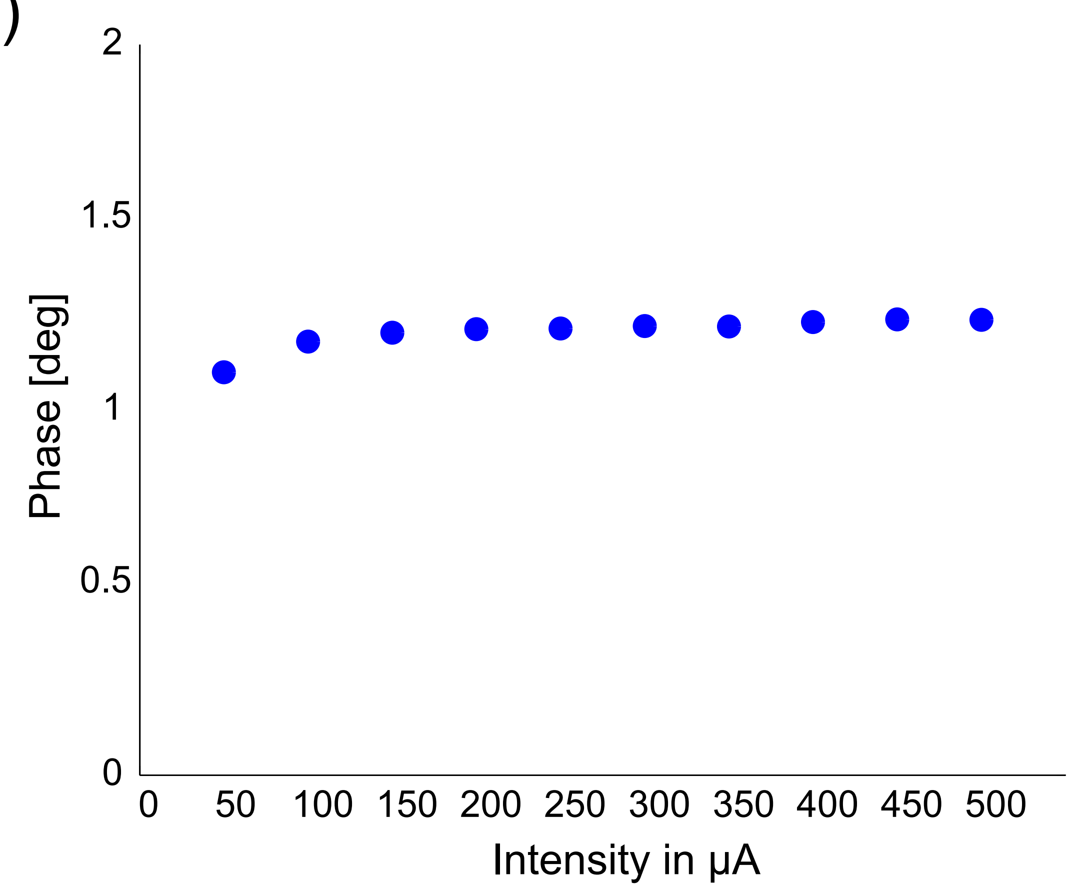
2
1.5
1
0.5
0
Phase [deg]
0
50
100
150
200
250
300
350
400
450
500
Intensity in µA

Supp Fig13

Monkey 1

A)

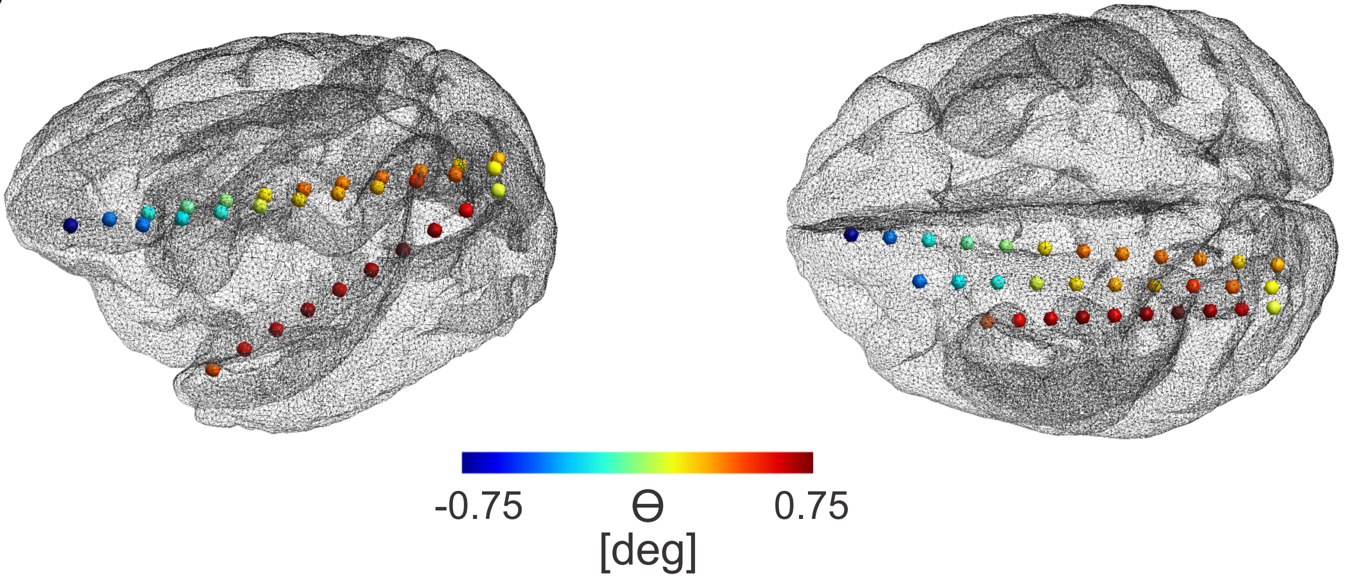

Monkey 2

B)

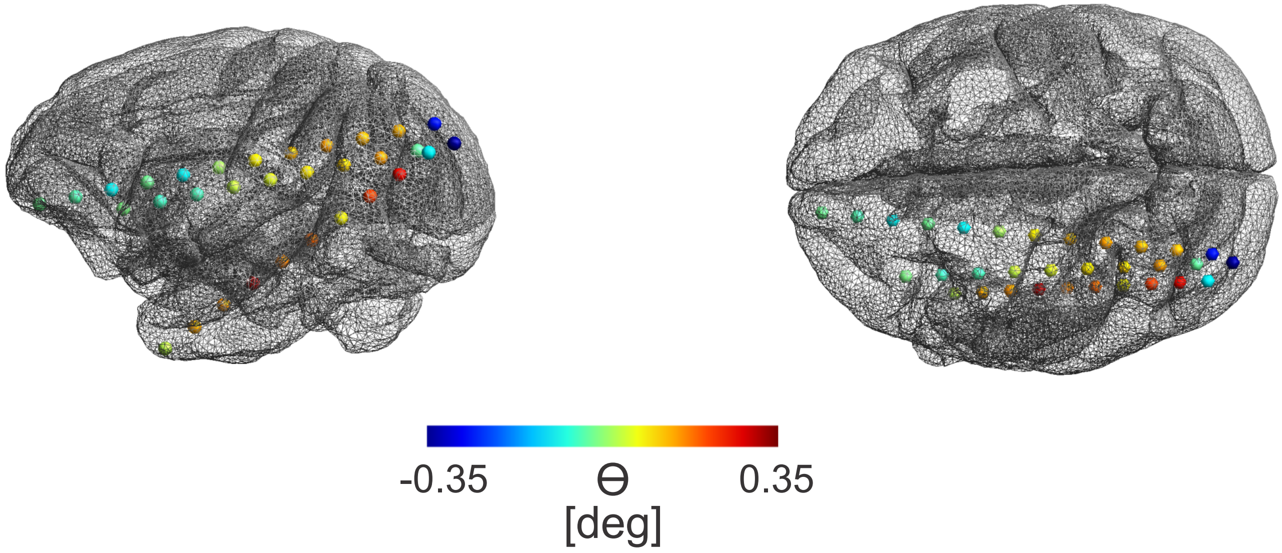

Supp Fig14

A)

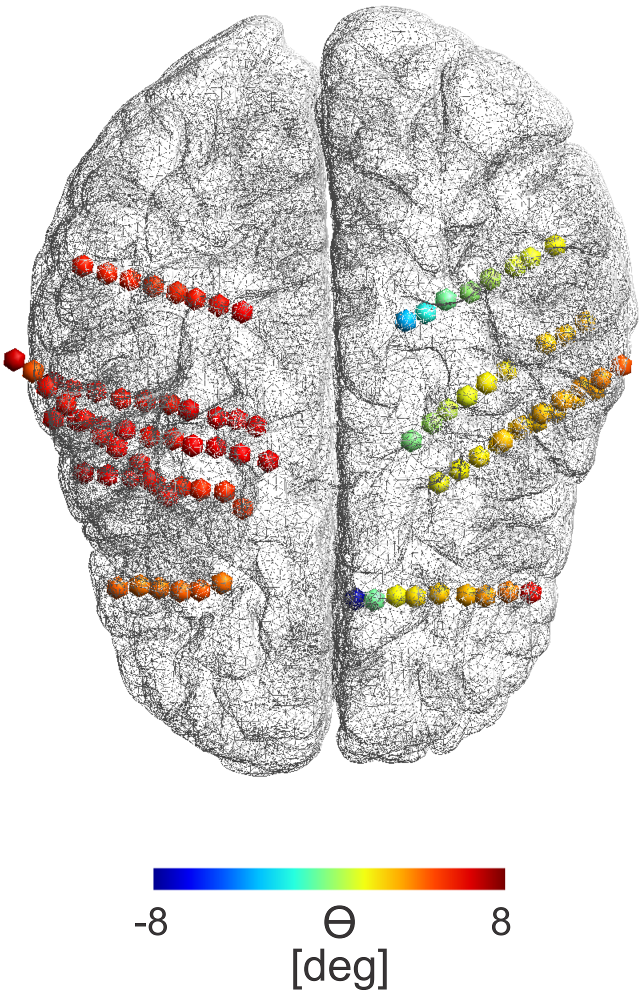

B)

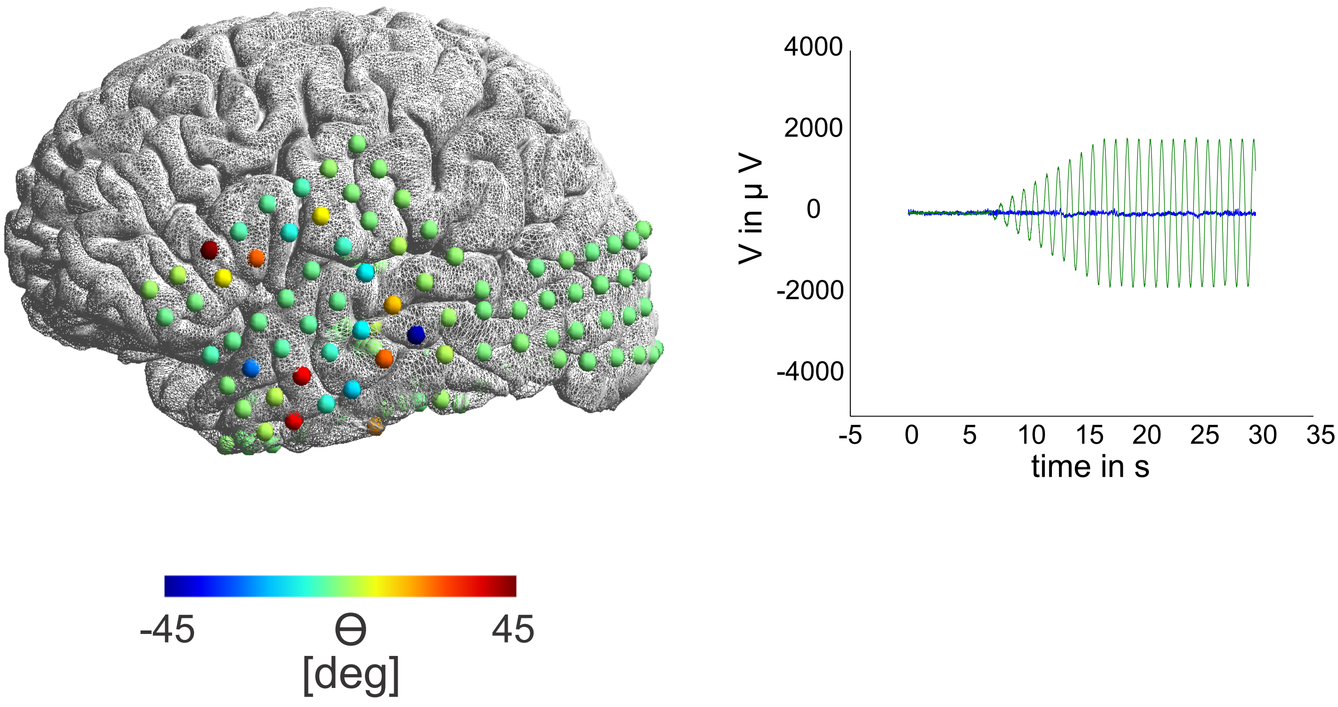

Supp Fig15

A)

B)

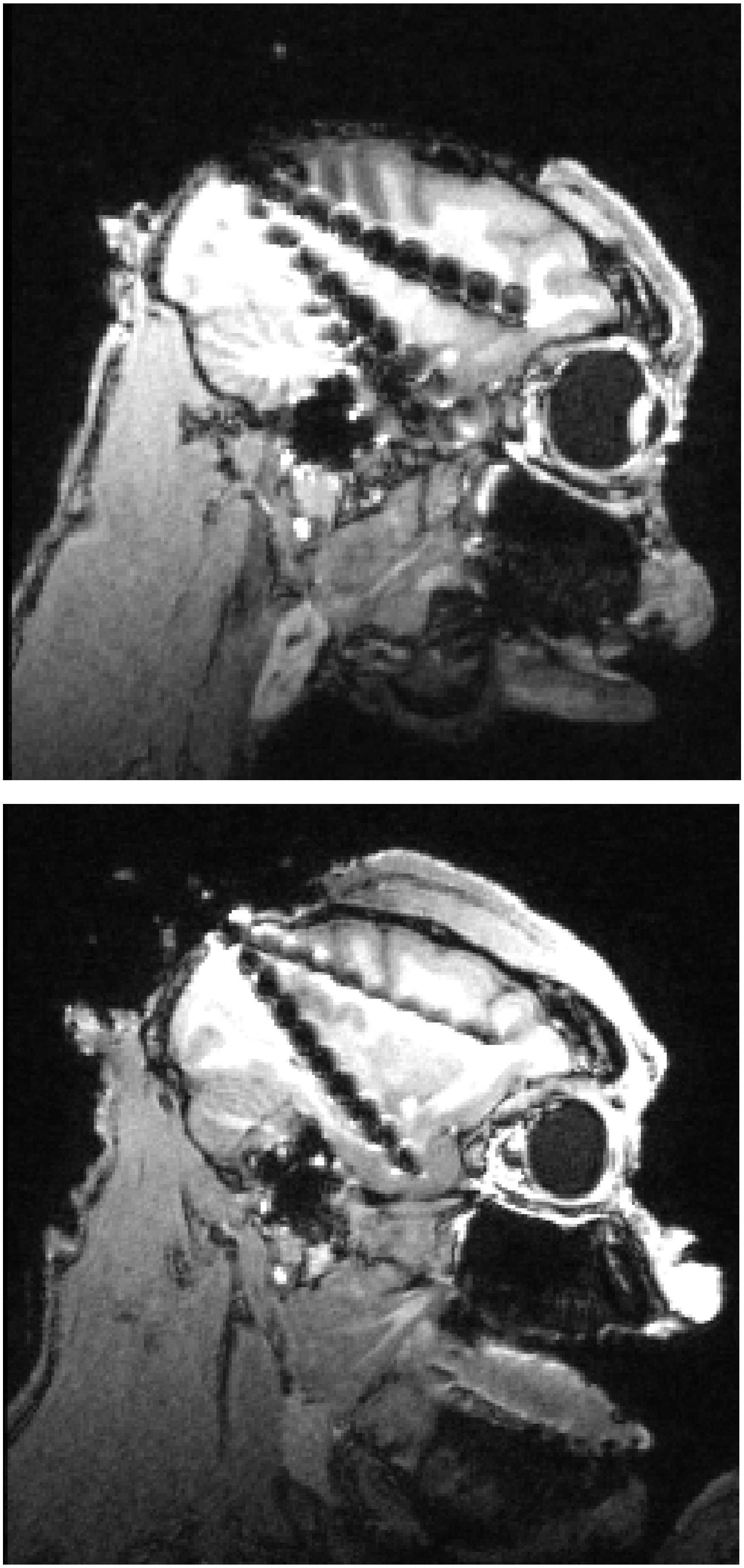

Supp Fig16

A)

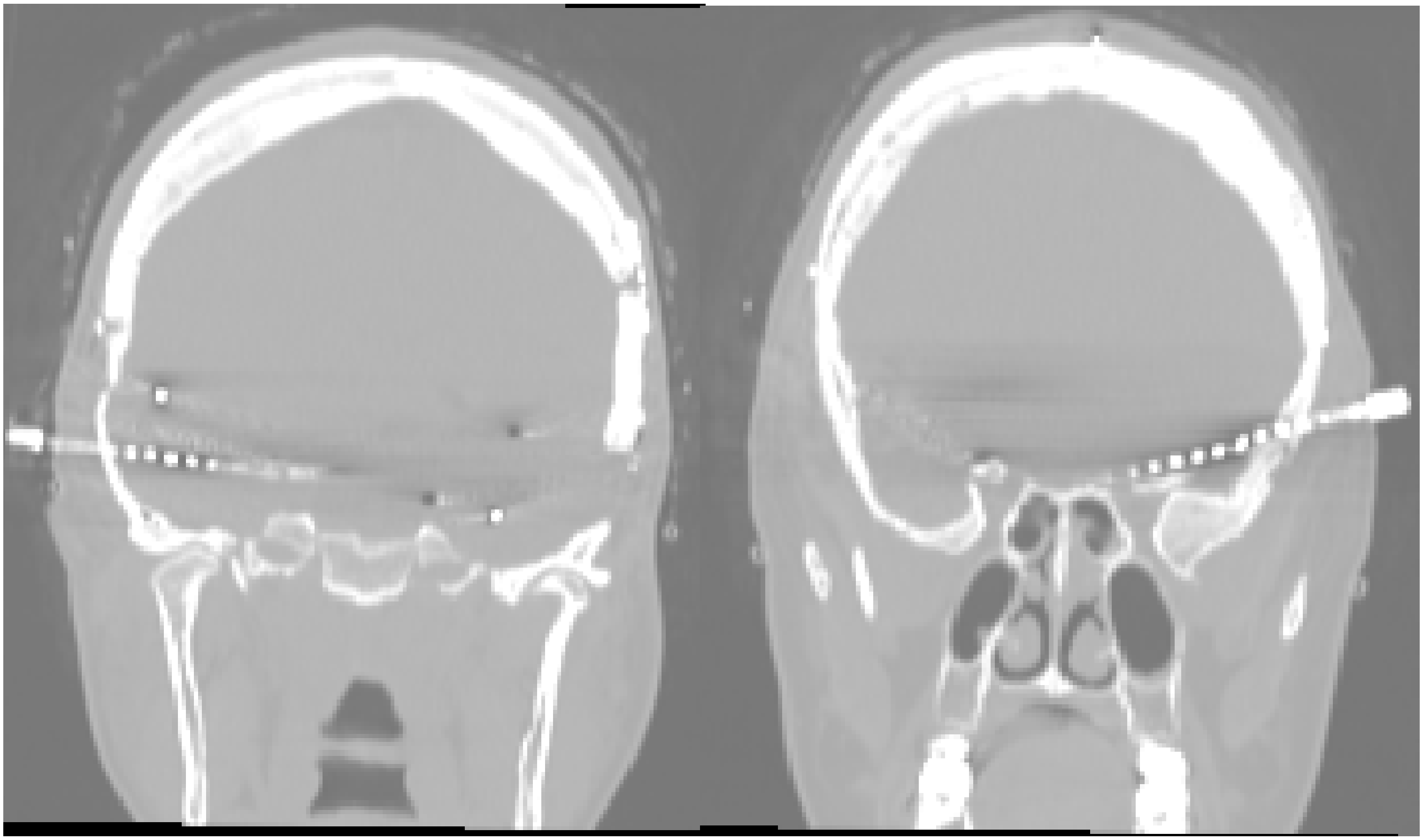

B)

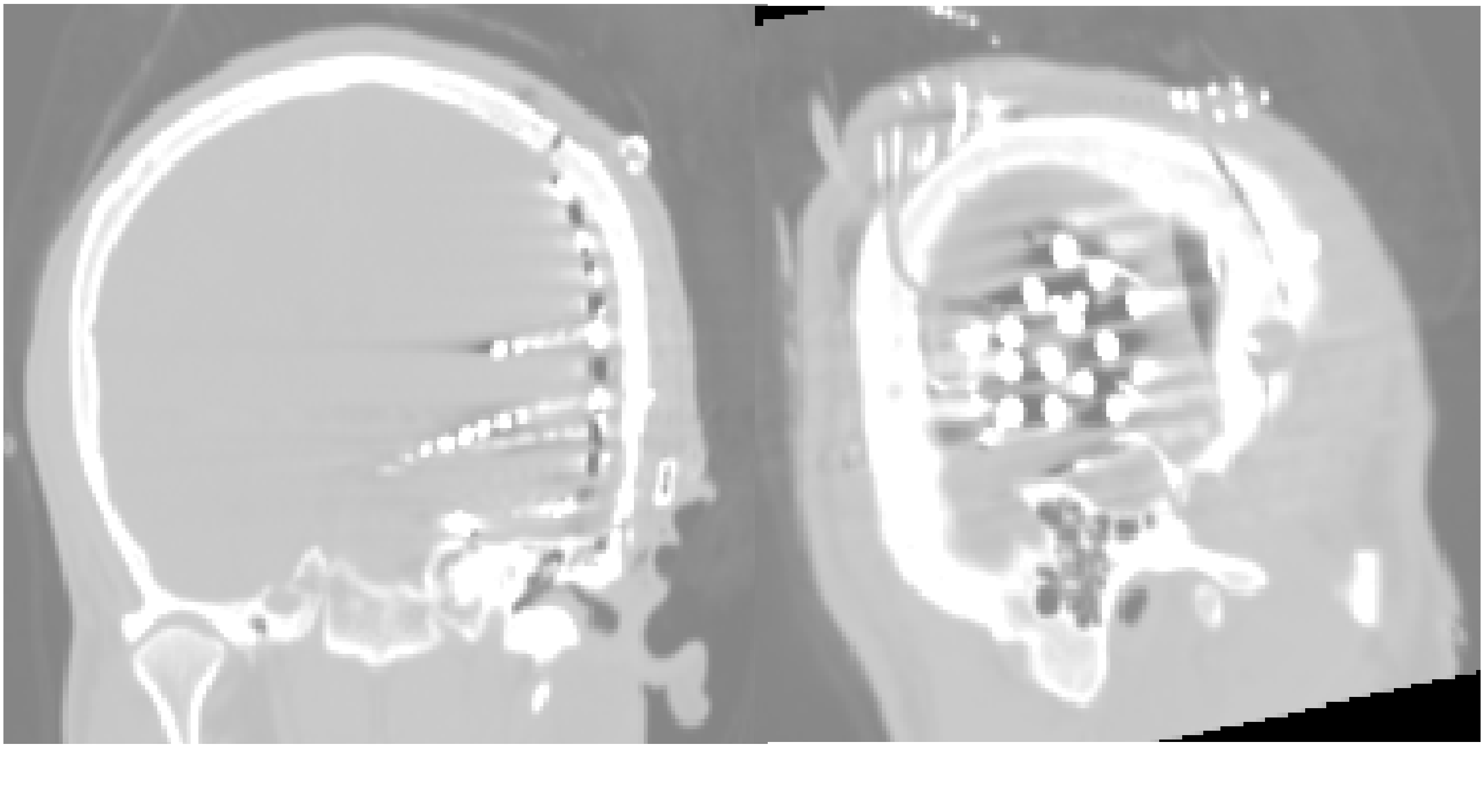

Supp Fig17

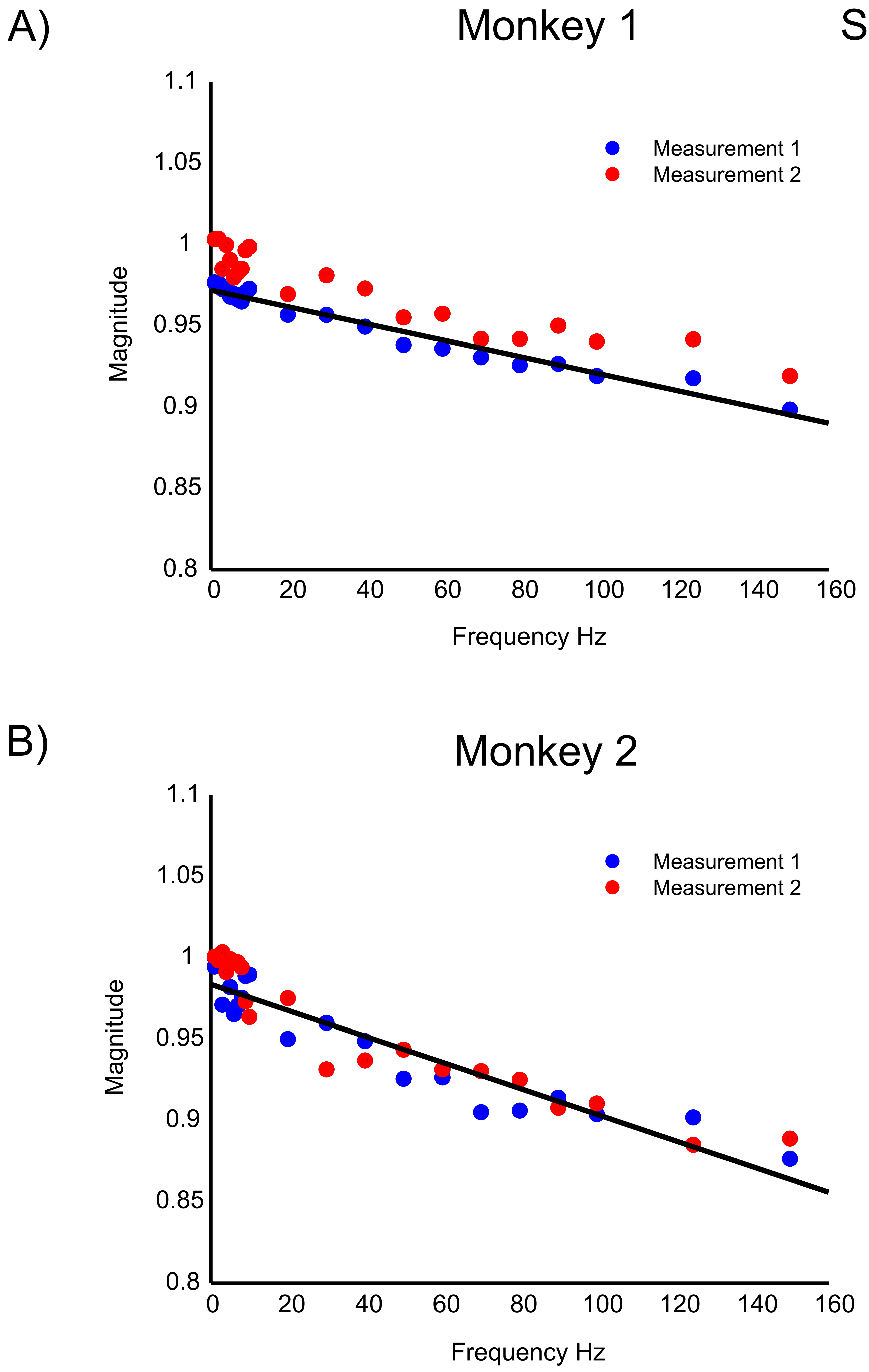
A)
Monkey 1
Measurement 1
Measurement 2
Magnitude
1.1
1.05
1
0.95
0.9
0.85
0.8
0
20
40
60
80
100
120
140
160
Frequency Hz
B)
Monkey 2
Measurement 1
Measurement 2
Magnitude
1.1
1.05
1
0.95
0.9
0.85
0.8
0
20
40
60
80
100
120
140
160
Frequency Hz
